# Treatment outcomes in pediatric empyema: a retrospective observational study from a tertiary center in Dubai, United Arab Emirates

**DOI:** 10.1007/s00383-025-06085-9

**Published:** 2025-06-12

**Authors:** Mohammed Al Blooshi, Munir Ahmad, Fadi Al Shamali, Wajeeh Uddin, Ghadir Jaber, Mamoun Al Marzouqi, Avinash Hiremath, Masih Abdul Kader, Vipul Gupta

**Affiliations:** Department of Pediatric Surgery & Urology, Al Jalila Children’s Specialty Hospital, 6th Street, Al Jaddaf, PO Box 300100, Dubai, United Arab Emirates

**Keywords:** Pediatric empyema, Fibrinolysis, Chest tube, Intrapleural therapy, Length of stay

## Abstract

**Purpose:**

To evaluate real-world effectiveness of intrapleural fibrinolysis versus drainage alone or surgery, and to identify factors linked to prolonged hospitalisation in children with pleural empyema at a tertiary centre. We also aimed to provide regional baseline data to guide future care.

**Methods:**

We retrospectively reviewed records for all patients aged ≤ 14 years treated for pleural empyema et al. Jalila Children’s Specialty Hospital, Dubai, from January 2021 to December 2024. Demographics, imaging, treatments, antibiotic use, and in-hospital outcomes were abstracted and summarised descriptively.

**Results:**

Thirty-five children (median age 4 years; 54% female) were included. All underwent ultrasound-guided tube thoracostomy, and 30 (86%) also received intrapleural alteplase. Four children needed surgical decortication and 1 child with lymphoma died. Median tube drainage was 6 days with fibrinolysis versus 8 days without. Median hospital stay was 11 days with fibrinolysis, 27 days without, and 13.5 days after surgery. Hospitalisation ≥ 15 days correlated with older age, omission of fibrinolysis, use of ≥ 4 antibiotics, and computed tomography imaging. No serious drug-related adverse events occurred.

**Conclusion:**

Prompt tube thoracostomy combined with intrapleural alteplase is a safe, effective, and resource-efficient first-line therapy for paediatric empyema, shortening hospital stay and markedly reducing the need for surgery.

## Introduction

This paper presents a retrospective observational study on the treatment outcomes of paediatric empyema at a tertiary centre, contributing local evidence to the global literature. Paediatric empyema—the accumulation of pus within the pleural space—most often arises as a complication of pneumonia [1, 2]. Bacterial invasion of pleural fluid triggers an inflammatory cascade through exudative, fibrinopurulent and organising phases, potentially leading to loculations and a restrictive pleural peel if treatment is delayed [1, 3]. Although the mortality rate is low in the modern era, paediatric empyema imposes a substantial acute-illness burden and often necessitates prolonged hospitalization [1].

### Background on paediatric empyema

Empyema accounts for an estimated 0.5–1% of childhood pneumonias, though this incidence fluctuates with pneumococcal conjugate vaccine (PCV) coverage [1]. Introduction of PCV7 initially reduced empyema cases, yet non-vaccine pneumococcal serotypes emerged [4]. Transition to PCV13, covering serotypes 1 and 3 among others, further lowered empyema hospitalisations in many regions [4]. *Streptococcus pneumoniae* remains the primary pathogen, with *Streptococcus pyogenes* and *Staphylococcus aureus* causing a smaller but significant proportion of cases [1, 2]. Shifts in pneumococcal serotypes continue to influence disease patterns, as evidenced by an Australian tertiary-centre report linking increased empyema incidence to serotype 3 [5]. These data underscore that paediatric empyema, while uncommon, remains an evolving clinical entity.

### Evolving treatment modalities

Management of paediatric empyema has progressed with improvements in antibiotic therapy, imaging and surgical techniques. Broad-spectrum intravenous antibiotics are initiated promptly [1]. Ultrasound-guided chest-tube placement is often employed for moderate or large collections; computed tomography (CT) scanning is reserved for complex presentations [1]. Most children improve within days when antibiotics are combined with adequate drainage.

When loculations or dense fibrinous adhesions impede fluid evacuation, intrapleural fibrinolysis or surgical intervention may be required. Fibrinolytic therapy agents such as urokinase or tissue plasminogen activator (tPA) via the chest tube to break down septations. Randomised trials have shown fibrinolysis can equal early surgery, including a landmark study by Sonnappa et al. [6] demonstrating equivalent outcomes between urokinase plus chest drainage and video-assisted thoracoscopic surgery (VATS). Marhuenda et al. [7] likewise reported no significant difference in hospital stay or treatment failure when comparing primary VATS with intrapleural urokinase.

Consequently, guidelines endorse either fibrinolysis or VATS as acceptable first-line options [6, 7]. VATS has replaced open thoracotomy in most centres thanks to reduced postoperative pain and faster recovery, yet the choice between early surgery and fibrinolysis often depends on local expertise and clinician preference [8]. Whichever strategy is used, close monitoring is essential so that, if one modality fails—e.g. inadequate drainage or persistent sepsis—timely conversion to the alternative can occur. Overall, advances in antibiotics, imaging and minimally invasive techniques have improved paediatric empyema outcomes, though high-level evidence on optimal timing and sequencing of interventions continues to evolve.

### Importance of local case series in empyema

Paediatric empyema presentation and management vary by region, pathogen distribution and resource availability, highlighting the importance of local case series. Much published evidence derives from high-income regions [8], which may not reflect outcomes where healthcare infrastructure or serotype prevalence differ. Single-centre studies offer detailed insight into real-world management, helping clinicians benchmark fever duration, length of stay (LOS) or intervention success against international norms while exposing unique regional challenges. This is especially relevant in resource-limited locations, where repeat fibrinolysis or early thoracoscopy may be cost-prohibitive [8]. Sharing local data therefore enriches the global evidence base by providing context-specific outcomes.

### Rationale for the present study

Al Jalila Children’s Specialty Hospital is a major tertiary referral centre in the Middle East, managing diverse paediatric infections, including empyema thoracis. Before this study, data on paediatric empyema outcomes from our region were scarce. Our retrospective review from June 2021 to December 2024 therefore aimed to characterise treatment outcomes, including patient demographics, imaging findings, use of fibrinolytics and need for surgery. We sought to assess how our predominantly non-operative approach performed and whether our results aligned with international benchmarks.

This investigation serves multiple purposes. First, it establishes baseline data on empyema cases in our patient population, pinpointing any delays or modifiable care factors specific to our setting. Second, it gauges the effectiveness of routine fibrinolysis in infection resolution, guiding local clinical decisions and resource allocation. Finally, by sharing these findings, we contribute a regional perspective on paediatric empyema to the broader literature, offering comparisons for other centres in similar environments. In essence, this study addresses the need for local evidence on paediatric empyema outcomes and seeks to inform and refine care both in our institution and beyond.

## Methodology

We conducted a retrospective observational study et al. Jalila Children’s Specialty Hospital in Dubai, United Arab Emirates. The study period spanned from January 2021 to December 2024. All patients aged 0–14 years who were admitted with a diagnosis of empyema during this interval were included.

Data were obtained from the hospital’s electronic medical records. For each eligible patient, we collected demographic information (age, sex), dates of admission and discharge, and the duration of symptoms prior to admission. We also recorded details of diagnostic imaging studies performed.

Treatment-related data included the initial antibiotic regimen and any use of intrapleural fibrinolytic therapy. We also recorded any surgical interventions, such as chest-tube placement or pleural decortication. Initial laboratory results at presentation, including inflammatory markers and cell counts, were documented for all patients.

## Results

### Patient characteristics

A total of 35 children were included in the study. The median age at presentation was 4 years (range 4 months–13 years). There were 16 males (46%) and 19 females (54%). All cases were diagnosed with pleural empyema; left-sided involvement was more common (23 cases, 66%) than right-sided (11 cases, 31%), and 1 patient (3%) had bilateral disease.

The median duration of symptoms before admission was 6 days (range 1–30 days). Approximately one-third of patients had significant underlying comorbid conditions (e.g. chronic neurologic or immunocompromising illnesses). Table 1 summarises the patient demographics and baseline clinical characteristics.

**Table 1:** Patient demographics and baseline characteristics

Characteristic	Value
Age (years), median (IQR)	4 [3–8]
Gender, n (%)	Male 16 (45.7%), Female 19 (54.3%)
Nationality, n (%)	UAE 13 (37.1%), Non-UAE 22 (62.9%)
Affected side, n (%)	Left 23 (65.7%), Right 11 (31.4%), Bilateral 1 (2.9%)
Symptom duration before admission (days), median (IQR)	6 [3–9]
Initial WBC (× 10^9/L), median (IQR)	17.0 [13.1–22.5]
Initial CRP (mg/L), median (IQR)	192 [120–290]
Initial PCT (ng/mL), median (IQR)	1.4 [0.6–5.4]

Values are presented as median [IQR] for continuous variables and n (%) for categorical variables. *WBC* white blood cell count, *CRP* C-reactive protein, *PCT* procalcitonin

### Treatment modalities and outcomes

All patients were managed with tube thoracostomy (chest-tube) drainage. Intrapleural fibrinolytic therapy with alteplase was administered in 30 patients (85.7%), while 5 patients (14.3%) did not receive fibrinolysis. The chest tube remained in place for a median of 6 days (range 2–18 days).

Four patients (11.4%) required escalation to surgical management. These procedures included 1 formal thoracotomy with decortication and 3 limited mini-thoracotomies for debridement or tube repositioning. One patient (2.9%) died during hospitalisation (a child with underlying lymphoma). The overall hospital LOS had a median of 12 days (range 6–132 days).

**Fig. 1:**
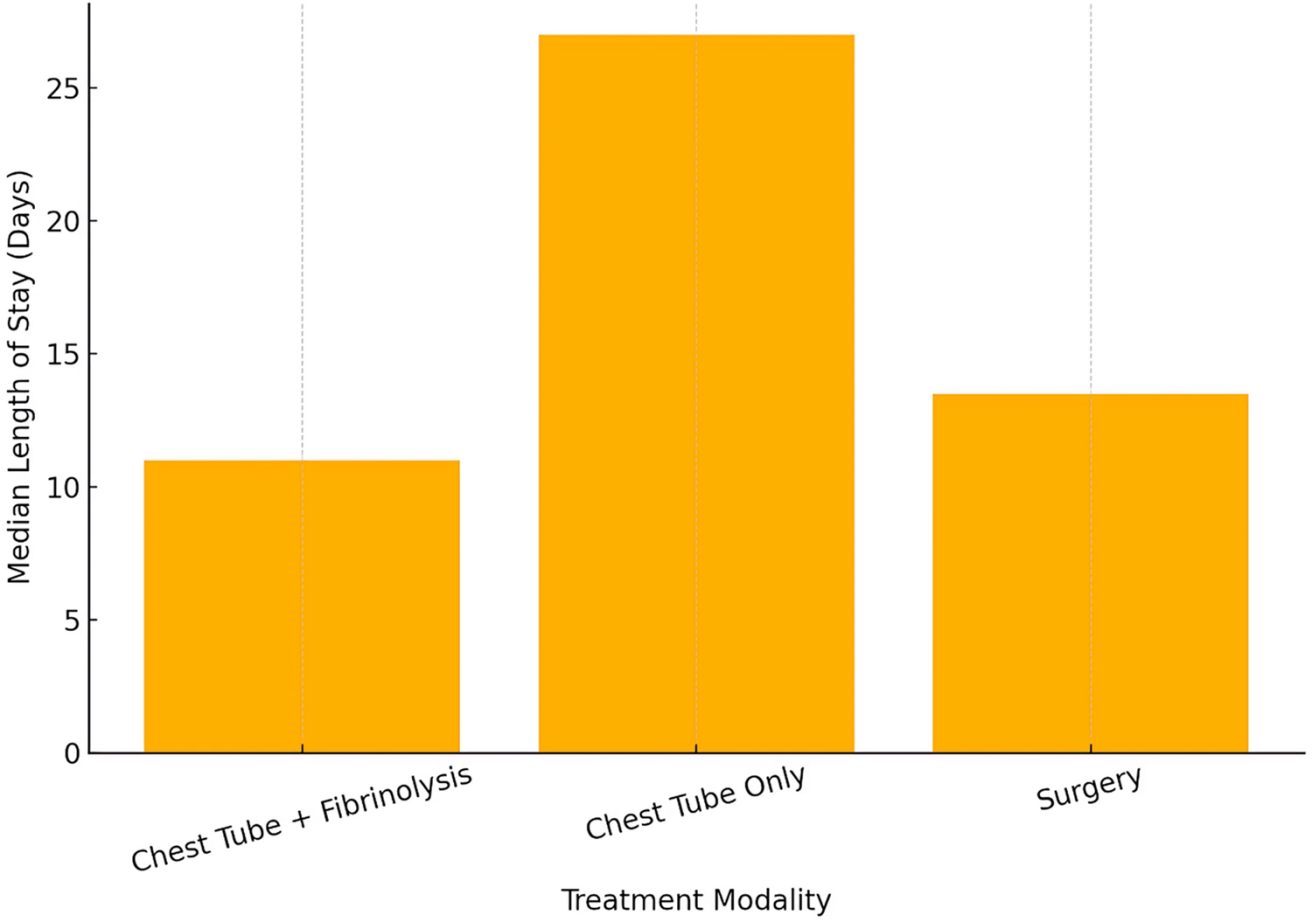
Median length of stay by treatment modality. Median length of hospital stay by treatment modality; vertical bars indicate the median days for children treated with chest-tube + fibrinolysis, chest-tube only and surgery, respectively; dotted grid lines are reference guides for the y-axis

Children managed with chest-tube drainage alone (no fibrinolytic) had the longest median LOS (27 days), compared with 11 days in those who received fibrinolysis and 13.5 days in those who required surgery (Fig. 1). Table 2 details the clinical interventions and outcomes stratified by treatment strategy.

**Table 2:** Clinical interventions and outcomes by treatment strategy

Variable	Fibrinolysis (n = 30)	No fibrinolysis (n = 5)
Chest tube duration (days), median (IQR)	6 [5–8]	8 [7–9]
Length of stay (days), median (IQR)	12 [9–14]	27 [22–27]
Surgical intervention required, n (%),median (IQR)	4 (13.3%)	0 (0%)

The fibrinolysis group received intrapleural alteplase. Chest-tube duration = days from insertion to removal; LOS = days from admission to discharge

### Imaging and surgical intervention

Chest ultrasound was utilised in all cases alongside chest radiography to confirm pleural-fluid collections and guide drainage. CT of the chest was performed in 9 patients (26%) who had either atypical presentations or inadequate response to initial management. Of the 9 children who underwent CT imaging, 3 (33%) ultimately required surgical decortication. In contrast, only 1 of 26 patients (4%) managed with ultrasound alone required surgery. Of the four surgical cases, three underwent limited ‘mini-thoracotomies’ and one had a formal thoracotomy. These approaches reflected surgeon preference rather than a lack of thoracoscopic expertise or resources. Surgery was undertaken when patients demonstrated failure of medical management (persistent fever, high inflammatory markers, inadequate drainage or reaccumulation on imaging).

Table 3 presents the relationship between imaging modality and the need for surgical intervention.

**Table 3:** Imaging modality vs. need for surgical intervention

Imaging modality	No surgical intervention, n (%)	Surgical intervention required, n (%)	Total (n)
CXR + US	25 (96.2%)	1 (3.8%)	26
CXR + US + CT	6 (66.7%)	3 (33.3%)	9

*CXR* chest radiograph, *US* ultrasound, *CT* computed tomography. Values are n (%) within each imaging category

### Antibiotic use and length of stay

All patients received broad-spectrum intravenous antibiotics. The most common empirical regimen was a combination of a third-generation cephalosporin (typically ceftriaxone) plus vancomycin, used in 28 cases (80%). Adjunctive azithromycin was given in 8 patients (23%), and 10 patients (29%) received linezolid. Four children (11%) required escalation to a carbapenem or other extended-spectrum agent owing to complicated infection.

Patients requiring these broader antibiotic regimens tended to have longer hospitalisations. For instance, 3 of the 4 children who received meropenem had hospital stays exceeding 3 weeks, whereas those treated with the standard ceftriaxone/vancomycin regimen typically had stays of only 1–2 weeks. Table 4 summarises the antibiotic regimens used and the associated LOS.

**Table 4:** Antibiotic regimens and length of stay

Antibiotic regimen	n (%)	Length of stay (days), median [IQR]
Monotherapy (single antibiotic)	2 (5.7%)	9.5 [8–11]
2–3 antibiotics	25 (71.4%)	11 [9–14]
≥ 4 antibiotics	8 (22.9%)	17.5 [13.8–27.0]

Monotherapy = single antibiotic. “ ≥ 4 antibiotics” = four or more different agents during admission (escalation or regimen changes). LOS values are median [IQR].

### Factors associated with prolonged hospitalisation

For analysis, prolonged hospital stay was defined as an admission lasting more than 14 days. Ten patients (28.6%) met this criterion. Several factors were more frequent in the prolonged-stay group. Underlying chronic medical conditions were present in 6 of the 10 patients (60%) with prolonged hospitalisation, compared with 4 of 25 (16%) in those with shorter stays. Lack of intrapleural fibrinolytic therapy was also associated with longer admissions: 4 of the 5 patients who did not receive fibrinolysis (80%) had a hospital stay > 14 days, versus 6 of 30 (20%) who received fibrinolytics. Additionally, 2 of the 4 patients (50%) requiring surgical intervention had prolonged stays, compared with 8 of 31 (26%) managed without surgery.

**Fig. 2:**
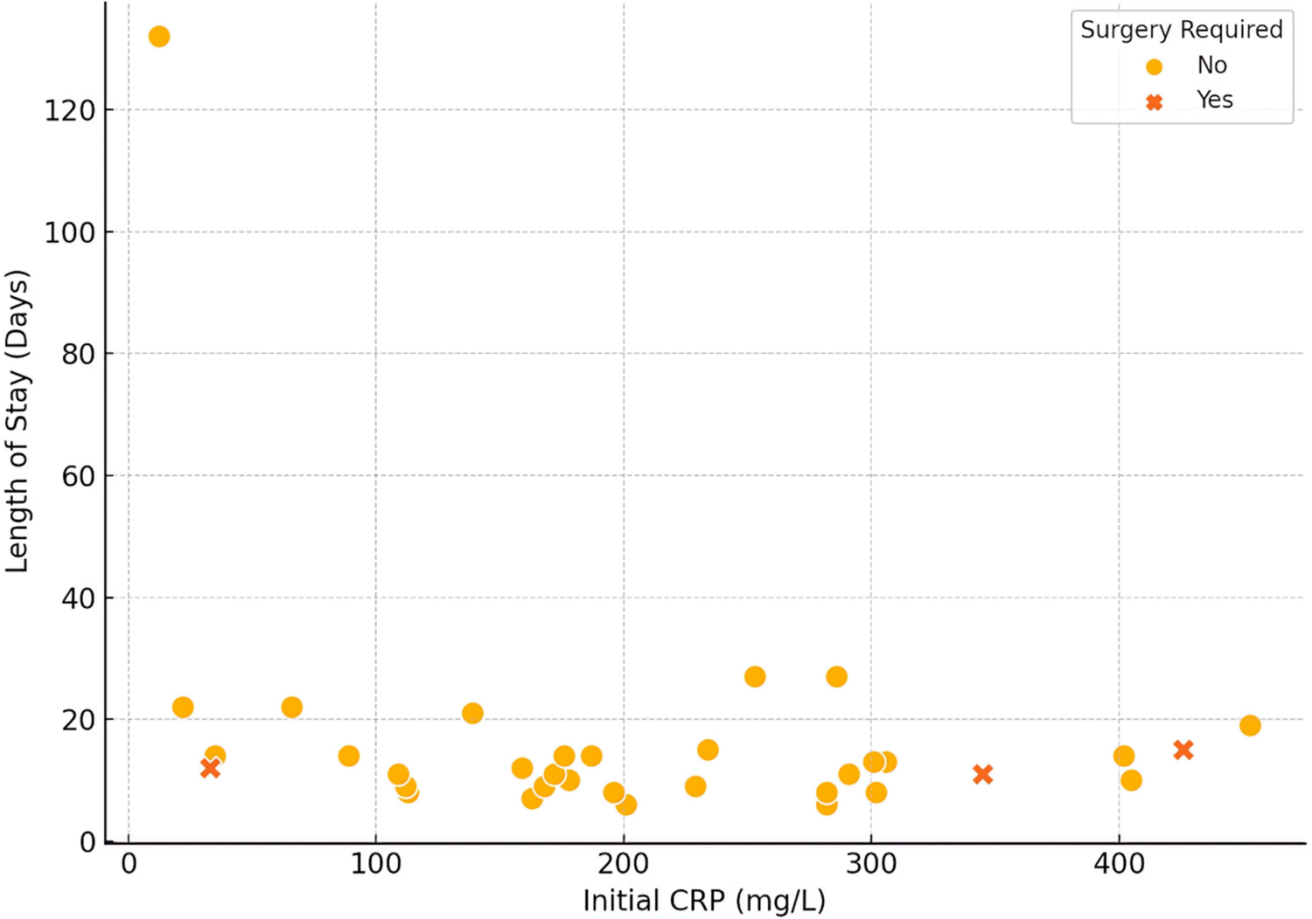
Initial CRP vs length of stay. Scatter-plot of initial C-reactive-protein concentration (x-axis) versus total hospital stay (y-axis); filled circles denote patients who avoided surgery and × symbols denote those who required surgery; grey grid lines and axis ticks mark increments of 50 mg L⁻.^1^ (x) and 20 days (y)

**Fig. 3:**
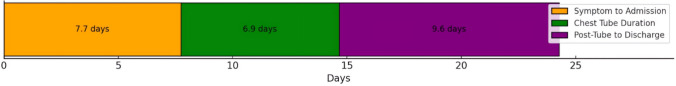
Average disease-course timeline (all patients combined). Stacked horizontal bar depicting the mean disease-course timeline; the orange segment shows days from symptom onset to admission, the green segment shows chest-tube duration and the purple segment shows the post-tube period until discharge; segment lengths are labelled with their corresponding mean values

There was no clear relationship between initial inflammatory-marker levels and LOS. In particular, initial C-reactive protein (CRP) values ranged widely and did not show a consistent correlation with duration of hospitalisation (Fig. 2). Table 5 outlines the key factors associated with prolonged stay. (Fig. 3) illustrates the average timeline of the disease course from symptom onset through intervention to recovery.

**Table 5:** Factors associated with prolonged hospital stay

Factor	< 15 days stay (n = 25)	≥ 15 days stay (n = 10)
Age (years), median (IQR)	3 [3–6]	7.5 [5.0–10.8]
Male, n (%)	13 (52%)	3 (30%)
Symptom duration (days), median (IQR)	6 [5–10]	2.5 [2.0–6.8]
Initial WBC (× 10^9/L), median (IQR)	20.0 [13.8–22.8]	15.9 [11.5–18.3]
Initial CRP (mg/L), median (IQR)	187 [159–291]	234 [66–286]
Initial PCT (ng/mL), median (IQR)	1.2 [0.6–4.8]	2.0 [0.4–5.6]
CT imaging performed, n (%)	5 (20%)	4 (40%)
Fibrinolysis given, n (%)	24 (96%)	6 (60%)
≥ 4 antibiotics used, n (%)	4 (16%)	4 (40%)
Surgical intervention required, n (%)	2 (8%)	2 (20%)

“Prolonged” stay = LOS ≥ 15 days (top quartile). *WBC* white-blood-cell count, *CRP* C-reactive protein, *PCT* procalcitonin, *CT* computed tomography.

## Discussion

This retrospective series of paediatric empyema cases shows that an initial non-operative strategy with chest-tube drainage plus intrapleural fibrinolysis can achieve excellent results. In our 35-patient cohort (2021–2024), > 90% were successfully managed without surgical decortication. Only three children (8.6%) required an open procedure (mini-thoracotomy or formal thoracotomy) after fibrinolysis, and no patient underwent video-assisted thoracoscopic surgery (VATS). These outcomes underscore that fibrinolytic therapy effectively resolved complicated pleural infections in most cases, with full recovery achieved via chest-tube drainage and alteplase instillation. No empyema-related deaths or major fibrinolytic complications occurred.

Our findings align with reports from other institutions. Gasior et al. [9] observed that most paediatric empyema cases can be “successfully treated without an operation,” with only ~ 16% eventually requiring VATS. Oyetunji et al. [10] went further by refining a protocol that emphasised prolonged fibrinolysis, reducing their decortication rate to 0% over time. They concluded that raising the threshold for surgery does not prolong length of stay (LOS) and can largely eliminate the need for operative intervention [10]. In our series, strict adherence to chest-tube placement, proper fibrinolytic dosing and close monitoring similarly made VATS unnecessary. Even the few surgical cases involved limited open debridement after an initial fibrinolysis attempt, highlighting that a well-structured, non-operative-first approach can minimise the need for surgical decortication.

Another key observation is that the LOS in our fibrinolysis-treated patients was comparable to international data. Our median LOS of about 12 days (interquartile range ≈9–15) aligns with other series. Oyetunji et al. [10] reported a median LOS of 8 days (interquartile range 6–11) using a fibrinolysis protocol, whereas Baram and Yaldo [11] noted a mean of 7.3 days in 95 children treated with tissue plasminogen activator (tPA). Although our LOS is slightly longer, this could reflect a few complex cases (including one child with significant comorbidities). Crucially, literature suggests that non-operative management does not inherently extend hospitalisation [10, 11]. In our practice, chest tubes were typically removed once drainage declined and clinical criteria improved (often within 4–10 days for uncomplicated cases). By discharge, children had defervescence and improved respiratory status, confirming that intrapleural therapy effectively resolved infection without faster surgical intervention.

Despite strong evidence favouring fibrinolysis, some authors still advocate primary VATS, citing potential faster recovery. A meta-analysis by Pacilli and Nataraja [12] suggested that VATS might slightly reduce re-intervention rates and shorten post-procedure LOS. However, overall complication rates between VATS and fibrinolysis were similar, and the authors noted that a few days’ difference in LOS may not outweigh the invasiveness and cost of surgery [12]. A recent network meta-analysis by Fernandez Elviro et al. [13] similarly found no major advantage in clinical outcomes among chest-tube drainage with fibrinolytics, VATS or open thoracotomy. Each intervention shortened stay more than antibiotics or chest tube alone, and fibrinolysis appeared to match VATS in efficacy while offering a cost benefit; short- and long-term morbidity were low across all methods [13]. Our findings reinforce the view that a well-executed fibrinolysis-based strategy typically matches VATS in success and timeline, but with reduced invasiveness, lower cost and no added complications.

Clinically, prioritising chest-tube drainage and fibrinolytic instillation means most paediatric empyema cases can be resolved without surgery. This approach is particularly advantageous in centres lacking robust paediatric thoracic surgical resources—effective fibrinolysis can prevent transfer or more aggressive intervention. Even where surgical expertise is available, reserving VATS for refractory cases spares children the pain and potential risks of an operation. In our series, complication rates from fibrinolysis were negligible: there were no serious bleeding events or adverse drug reactions, consistent with other reports of tPA safety [11]. The fact that only three patients required surgery further supports the value of persisting with non-operative measures before resorting to decortication. Similar to Oyetunji et al. [10], we found that “pushing further” with intrapleural therapy was typically rewarded with clinical improvement. Naturally, vigilance is essential, and surgery should be pursued without hesitation if clinical deterioration occurs or the empyema cannot be drained adequately.

### Limitations

As a retrospective single-centre study, our findings could be influenced by selection bias or undocumented confounders. We lack a randomised comparison to VATS or open surgery. Although following a uniform protocol internally is a strength, it precludes direct comparisons of different treatment strategies within our dataset. Moreover, our results might not fully generalise to lower-volume centres or populations with different demographics. Inherent to retrospective design, missing or inconsistently recorded data are also a possibility. We focused on in-hospital outcomes, with limited long-term follow-up. While previous research suggests most children regain normal lung function after empyema [11], we cannot exclude subtle long-term differences in our cohort. Finally, our sample of 35 cases is modest, limiting statistical power for subgroup analyses or rare complications. Larger multicentre studies could validate and expand upon our findings. 

## Conclusion

Our experience corroborates extensive evidence that chest tube plus intrapleural fibrinolysis is a highly effective first-line therapy for paediatric empyema. Success rates matched those reported internationally, and LOS was similar to or slightly longer than typical VATS timelines, without any clear clinical disadvantage. With comparable efficacy to surgery and a lower invasiveness and cost burden, fibrinolysis can be the default management. Only a small subset of children required open surgery, reflecting its role as a valuable backup rather than a primary approach. These results affirm current guidelines endorsing either fibrinolysis or VATS and strengthen the argument for non-operative management in the majority of paediatric empyema cases. By offering excellent recovery rates, minimal morbidity and cost-effectiveness, fibrinolytic therapy aligns well with patient-centred care. Future prospective comparisons, ideally randomised and multicentre, will further delineate long-term outcomes and refine best-practice algorithms. In the meantime, this study provides reassurance that an “initial fibrinolysis, conditional surgery” paradigm is safe, effective and beneficial for children, especially in tertiary centres able to monitor clinical response and intervene surgically if needed.

## Data Availability

The datasets generated during and/or analysed during the current study contain confidential patient information and are not publicly available. De-identified data may be shared by the corresponding author on reasonable request and with prior Institutional Review Board approval.
